# SerpinA3N deficiency deteriorates impairments of learning and memory in mice following hippocampal stab injury

**DOI:** 10.1038/s41420-020-00325-8

**Published:** 2020-09-18

**Authors:** Zhi-Meng Wang, Cong Liu, Ying-Ying Wang, Yu-Sen Deng, Xuan-Cheng He, Hong-Zhen Du, Chang-Mei Liu, Zhao-Qian Teng

**Affiliations:** 1 State Key Laboratory of Stem Cell and Reproductive Biology, Institute of Zoology, Chinese Academy of Sciences, 100101 Beijing, China; 2 Savaid Medical School, University of Chinese Academy of Sciences, 100408 Beijing, China; 3 Institute for Stem Cell and Regeneration, Chinese Academy of Sciences, 100101 Beijing, China

**Keywords:** Cell death in the nervous system, Learning and memory

## Abstract

Traumatic brain injury is a global leading cause of disability and death, which puts patients at high risk for developing dementia. Early intervention is believed as the key to minimize the development of brain damages that could aggravate the symptoms. Here, we report that the serine protease inhibitor SerpinA3N is upregulated in hippocampal neurons in the early stage of hippocampal stab injury (HSI), while its deficiency causes a greater degree of neuronal apoptosis and severer impairments of spatial learning and memory in mice after HSI. We further show that MMP2 is a key substrate of SerpinA3N, and MMP2 specific inhibitor (ARP100) can protect against neuronal apoptosis and cognitive dysfunction in mice after HSI. These findings demonstrate a critical role for SerpinA3N in neuroprotection, suggesting that SerpinA3N and MMP2 inhibitors might be a novel therapeutic agents for neurotrauma.

## Introduction

Traumatic brain injury (TBI) is a global leading cause of disability and death across all ages, especially among children and young adults, with long-term devastating consequences on the patients^1^. The pathophysiological process of TBI can be divided into primary mechanical injury and delayed secondary injury^2–4^. Primary includes concussion, contusion, laceration, and diffused axonal injury, and secondary injury occurs within hours to days after brain injury, involving a series of pathological reactions, such as inflammation, oxidative stress, mitochondrial failure, and apoptosis^5–8^. While there has been significant improvement in reducing TBI-related mortality in the past decade, there are still no effective therapeutic strategies in overcoming long-term deficits involving sensory motor and memory functions.

TBI can lead to permanent dysfunctions or temporary damage that induce severe cognitive, physical, and emotional disturbances^9,10^. Early intervention is believed as the key to minimize the development of brain damages that could aggravate the symptoms^11^. Serine protease inhibitors (serpins) belong to protease inhibitor superfamily and are involved in various physiological and pathological processes, including immune response, blood coagulation, complement formation, cell migration and differentiation, hormone formation and transport, cell matrix reconstruction, blood pressure regulation, and intracellular protein hydrolysis, etc. SerpinA3N is the mouse orthologue of human α-1-antichymotrypsin (ACT) and its overexpression of SerpinA3N has been shown to accelerate wound healing in diabetic skin ulcers, to reduce neuropathic pain by inhibiting the activity of Granzyme B (GrB), and to mitigate myofiber degeneration^12–14^. Moreover, knockout of *Serpina3n* develops more neuropathic mechanical allodynia^15^. SerpinA3N is highly expressed in brain^16^, however, its roles in neurological diseases are still unknown.

In this study, we generated a mouse model of hippocampal stab injury (HSI) and evaluated whether SerpinA3N plays a role in HSI, and if targeting SerpinA3N pathway leads to cognitive recovery after HSI. Here, we found that SerpinA3N was upregulated in hippocampal neurons only in the early stage of HSI. Mice lacking *Serpina3n* displayed a greater degree of neuronal apoptosis and severer impairments of spatial learning and memory after HSI. We identified MMP2 as a key downstream target of SerpinA3N and provided evidence showing that ARP100, an MMP2 specific inhibitor, could reduce neuronal apoptosis and enhance cognitive function recovery after HSI.

## Results

### SerpinA3N is upregulated in neurons in the early stage of hippocampal stab injury

**Fig. 1:**
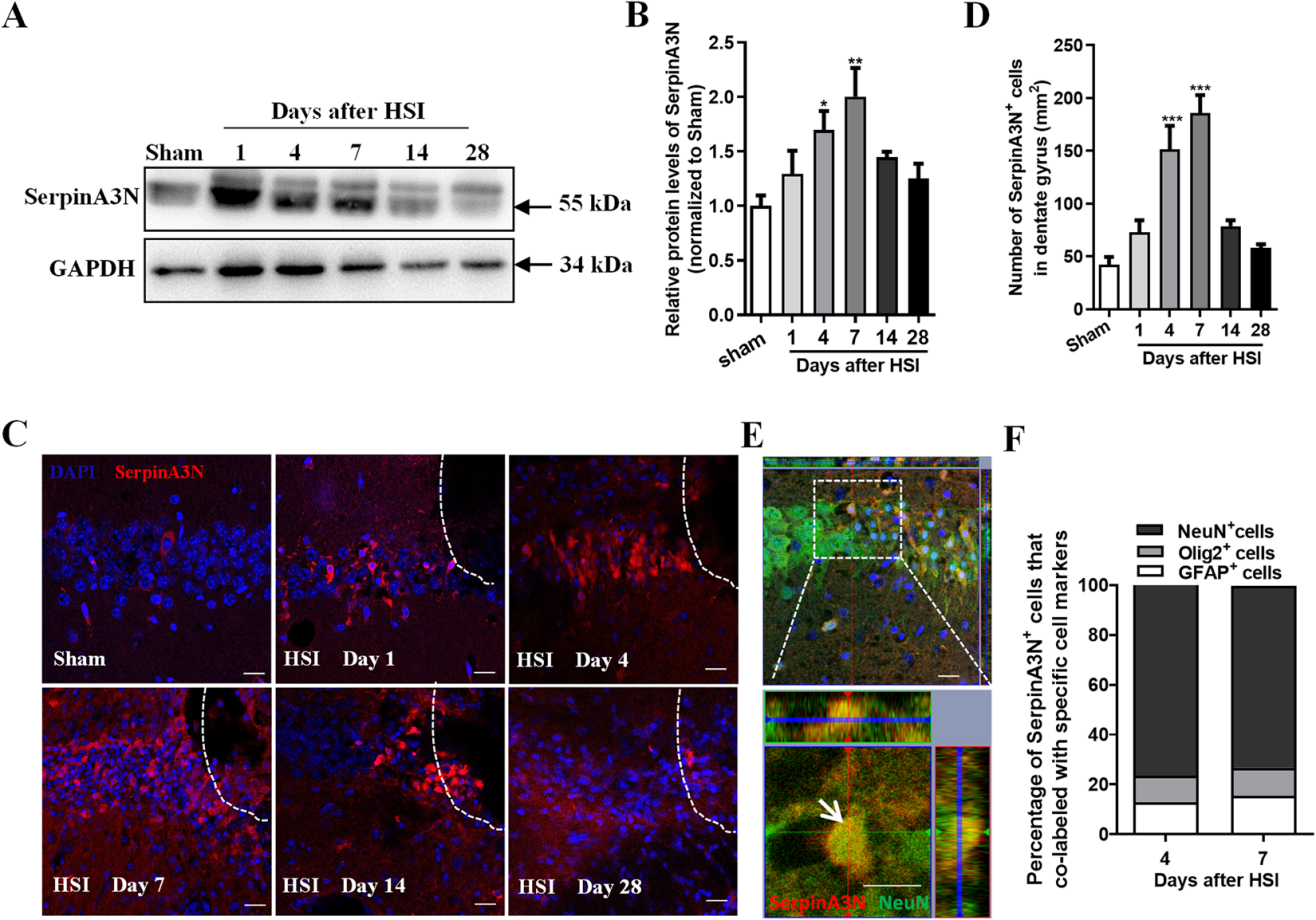
SerpinA3N is highly expressed in neurons in the early stage of hippocampal stab injury. **a**, **b** Representative image of western blot and Western blot analysis demonstrated that the protein expression of SerpinA3N was significantly increased in the early stage (Day 4 and Day 7) of hippocampal stab injury (HSI) and gradually declined to normal levels afterwards (*n* = 4). GAPDH was used as a loading control, and the protein expression levels of SerpinA3N were quantified by normalizing against GAPDH. **c** Representative images of SerpinA3N (red) immunostaining in the hippocampus at different time points following HSI. **d** Quantification analysis exhibited that SerpinA3N-positive cells dramatically increased in the early stages (Day 4 and Day 7) of HSI compared to sham treatment (*n* = 3). **e** Representative images of the co-staining of SerpinA3N and the neuron marker NeuN at 4 days after HSI. Arrow indicates the colocalization of SerpinA3N and NeuN. Scale bars, 20 μm. **f** Quantitative analysis showed that the majority of SerpinA3N^+^ cells (around 80%) were neurons, only a small amount of SerpinA3N^+^ cells were oligodendrocytes and astrocytes the early stages (Day 4 and Day 7) of HSI (*n* = 5). Data are represented as the mean ± SEM; two-tailed *t*-test, *p < 0.05, **p < 0.01, ***p < 0.001.

To examine the expression of SerpinA3N following traumatic brain injury, we established a hippocampal stab injury (HSI) model and detected SerpinA3N protein levels in the injured hippocampus at different time points after HSI by using Western blotting and immunostaining. Western blot assay demonstrated that SerpinA3N protein expression levels were significantly upregulated in the early stage (Day 4 and Day 7) of HSI and subsequently declined to normal levels afterward (Fig. 1a). Immunostaining analysis of SerpinA3N found that SerpinA3N positive cells dramatically increased in the injured hippocampus in the early stage (Day4 and Day7) of HSI (Fig. 1b, c).

Next, we performed immunofluorescence double staining of SerpinA3N and neuronal marker NeuN, astrocytic marker GFAP, microglial marker Iba1, or anti-oligodendrocyte marker Olig2 in hippocampus at 4 days after HSI. We observed that the majority of SerpinA3N^+^ cells (79.57%) were neurons, only a small amount of SerpinA3N^+^ cells were oligodendrocytes and astrocytes, but no SerpinA3N^+^ cells were microglia (Fig. 1d, e and Supplementary Fig. 1a).

To further validate the activation of SerpinA3N in neurons, we isolated primary hippocampal neurons and treated with different stimuli. Both SerpinA3N mRNA and protein levels were significantly elevated in cultured hippocampal neurons incubated with condition medium from BV2 cells subjected to LPS exposure (Supplementary Fig. 1b–d). Taken together, these findings suggested that SerpinA3N is upregulated in neurons only in the early stage of HSI. Increased SerpinA3N levels indicated that SerpinA3N may play a role in neurons after HSI.

### Serpina3n deficiency resulted in more neurons underwent apoptosis in response to hippocampal stab injury

**Fig. 2:**
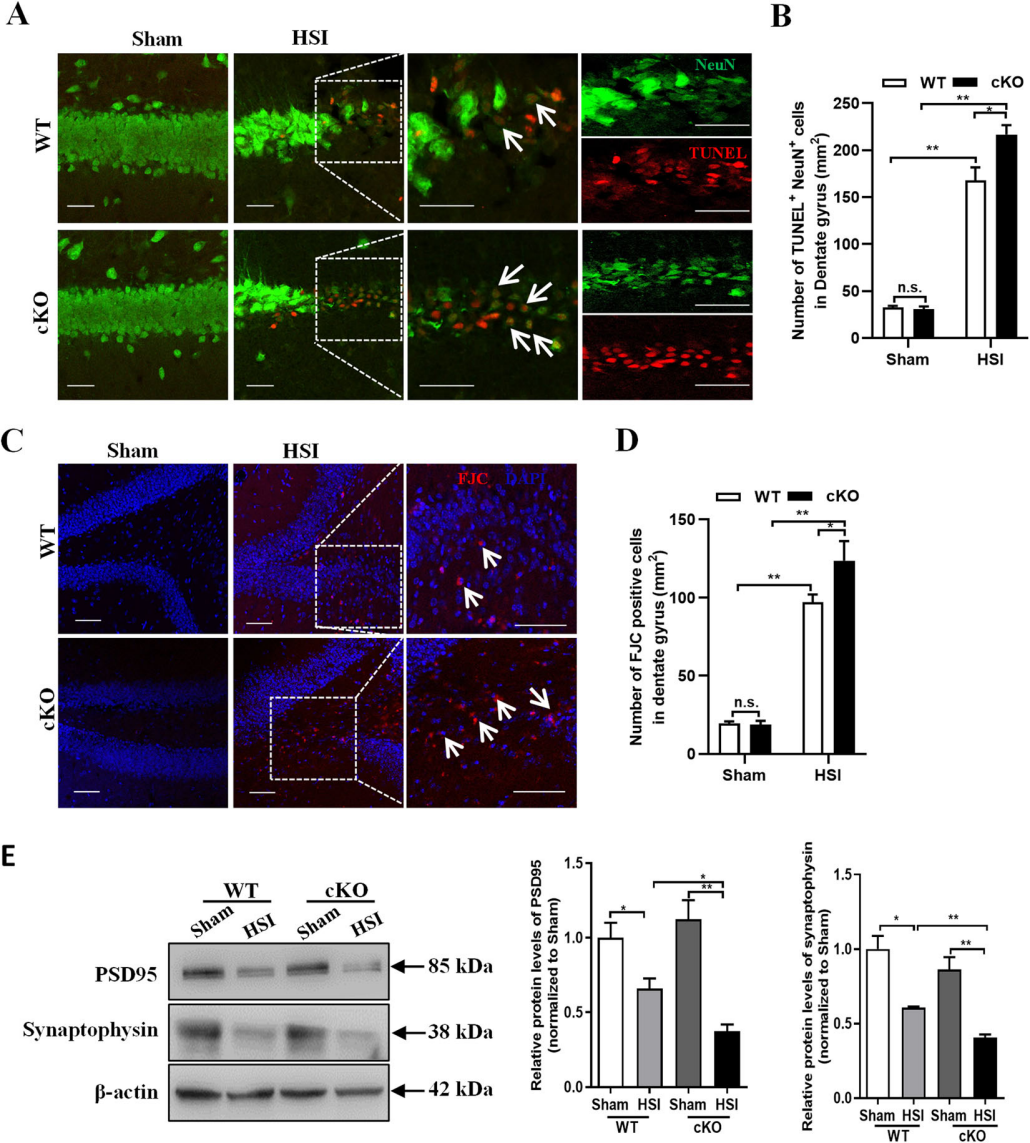
Knockout of Serpina3n results in more neurons underwent apoptosis in response to hippocampal stab injury. **a**, **b** Representative images and quantification of TUNEL (red) staining in the hippocampus from *Serpina3n* WT and cKO mice at 4 days after HSI. Compared with WT mice, *Serpina3n* cKO mice showed more neurons underwent apoptosis in response to HSI (*n* = 3). Scale bars, 20 μm. **c**, **d** Representative images and quantification of FJC (red) staining in the hippocampus confirmed that more apoptotic neurons existed in *Serpina3n* cKO hippocampus at 4 days post HSI (*n* = 3). Scale bars, 50 μm. **e** Western Blot analysis demonstrated that the expression levels of PSD95 and Synaptophysin were significantly lower in the injured hippocampus of *Serpina3n* cKO mice than that of WT mice (*n* = 3). β-actin was used as an endogenous control for protein expression. Data are represented as the mean ± SEM; two-tailed *t*-test, *p < 0.05, **p < 0.01, ***p < 0.001.

In order to determine the role of SerpinA3N in neurons, we firstly quantified TUNEL^+^ and NeuN^+^ cells in both the contralateral and ipsilateral hippocampus of *Serpina3n* WT and cKO mice at 4 days after HSI. In the ipsilateral hippocampus adjacent to the lesion site, more TUNEL^+^NeuN^+^ cells were observed in *Serpina3n* cKO mice than that of *Serpina3n* WT mice, whereas no significant difference was found in the contralateral hippocampus between *Serpina3n* cKO and WT mice (Fig. 2a, b). To further investigate whether *Serpina3n* deletion could affect progressive neurodegeneration after HSI, we then conducted FJC staining in the hippocampus and assessed the number of FJC^+^ neurons. Quantitative results confirmed that a significant increase in the number of degenerating neurons in *Serpina3n* cKO hippocampus at 4 days after HSI (Fig. 2c, d). We then investigated whether the loss of *Serpina3n* could influence the expression levels of synaptic proteins after HSI. Western blot analysis showed that PSD95 and synaptophysin protein levels were significantly decreased in the injured hippocampus of *Serpina3n* cKO mice than that of WT controls (Fig. 2e). Taken together, these results demonstrated that *Serpina3n* deficiency leads to more neurons underwent degeneration after HSI and suggested that SerpinA3N may serve as a neuroprotector after HSI.

### Serpina3n knockout resulted in severer impaired learning and memory in mice after hippocampal stab injury

**Fig. 3:**
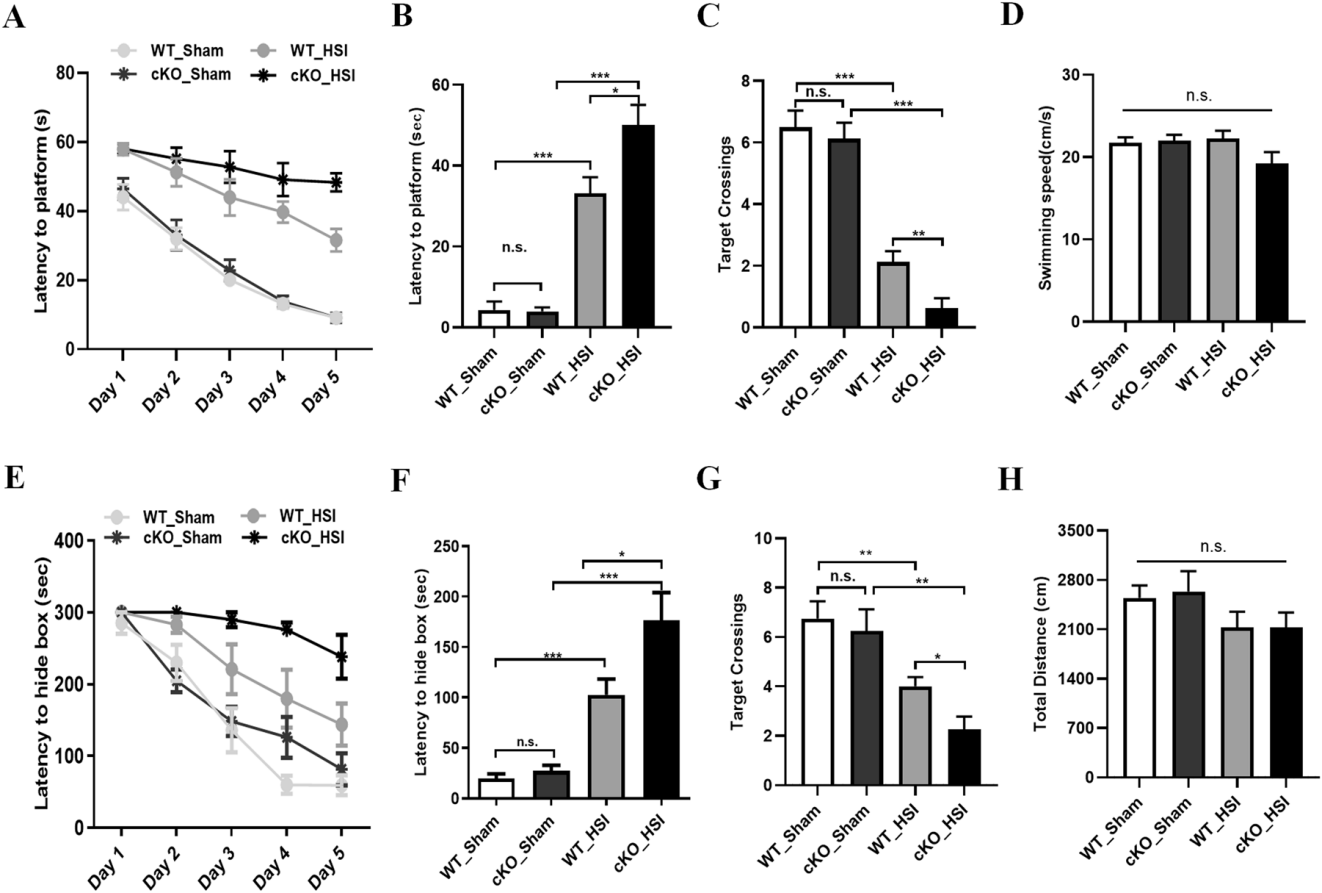
Serpina3n knockout resulted in impaired spatial learning and memory in mice after hippocampal stab injury. **a** Morris water maze test was started to evaluate the spatial learning and memory of *Serpina3n* WT and cKO mice at 10 days postHSI. During the training phase, each group showed improved latency to locate the platform, but compared with sham groups, both hippocampus-injured groups exhibited a significant delay to locate the platform. Moreover, injured *Serpina3n* cKO mice spent longer time to find the platform than injured WT mice (*n* = 8 mice per group). **b** In the probe test, both injured *Serpina3n* WT and injured cKO mice displayed a significantly longer latency to locate the platform compared with their sham controls, and injured cKO mice spent a longer time to locate the target compared to injured WT mice (*n* = 8 mice per group). **c** Injured *Serpina3n* cKO mice had fewer target crossings than injured WT mice in the probe test (*n* = 8 mice per group). **d** In the probe test, no significant difference in the swimming speed was observed between all the groups (*n* = 8 mice per group). **e** During the training phase of Barnes maze test, mice in every group showed improved latency of first entrance into the hiding box, but injured mice spent more time reaching the hiding box compared with their sham controls, while injured *Serpina3n* cKO mice spent the longest time to locate the hiding box (*n* = 8 mice per group). **f** In the probe test, both injured *Serpina3n* WT and injured cKO mice had a significantly longer latency to locate the hiding box compared with their sham controls, and injured cKO mice spent a longer time to reach the target compared to injured WT mice (*n* = 8 mice per group). **g** Injured *Serpina3n* cKO mice had fewer target crossings than injured WT mice in the probe trials of Barnes maze test (*n* = 8 mice per group). **h** No significant difference in total moving distance was detected between all different groups in the probe trials of Barnes maze test (*n* = 8 mice per group). Data are represented as the mean ± SEM; *p < 0.05, **p < 0.01, ***p < 0.001.

Next, we sought to determine whether *Serpina3n* loss-of-function would impair the spatial learning and memory in mice after HSI. The Morris water maze test was performed at 10 days postHSI to evaluate the spatial learning and memory. After HSI, both injured *Serpina3n* WT and injured cKO mice showed a significant delay in locating the platform in the training trails. However, injured *Serpina3n* cKO mice took a longer time to reach the platform than injured WT mice (Fig. 3a). In the subsequent probe test, *Serpina3n* cKO mice exhibited severer spatial learning and memory impairment with a significant higher latency to locate the platform and fewer target crossings, but did not affect the swimming speed compared to WT controls after HSI (Fig. 3b, c). No statistically significant difference in the swimming speed was observed between all the groups (Fig. 3d), suggesting that the different latency to locate the platform and the different target crossings were not due to differences in the speed of swimming but rather to different learning and memory processes among the groups.

To further assess the impaired cognitive function of *Serpina3n* cKO mice, we preformed Barnes maze test and also found that injured *Serpina3n* cKO mice spent more time reaching the hiding box, and they had fewer target crossings than injured WT mice both in the training phase and in the probe test phase (Fig. 3e–g). Total moving distance was not significantly different between the four groups (Fig. 3h). These behavioral data strongly support the idea that that Serpina3n plays a key role in neuroprotection against cognitive dysfunction after HSI.

### MMP2 is a substrate of SerpinA3N in the hippocampus

**Fig. 4:**
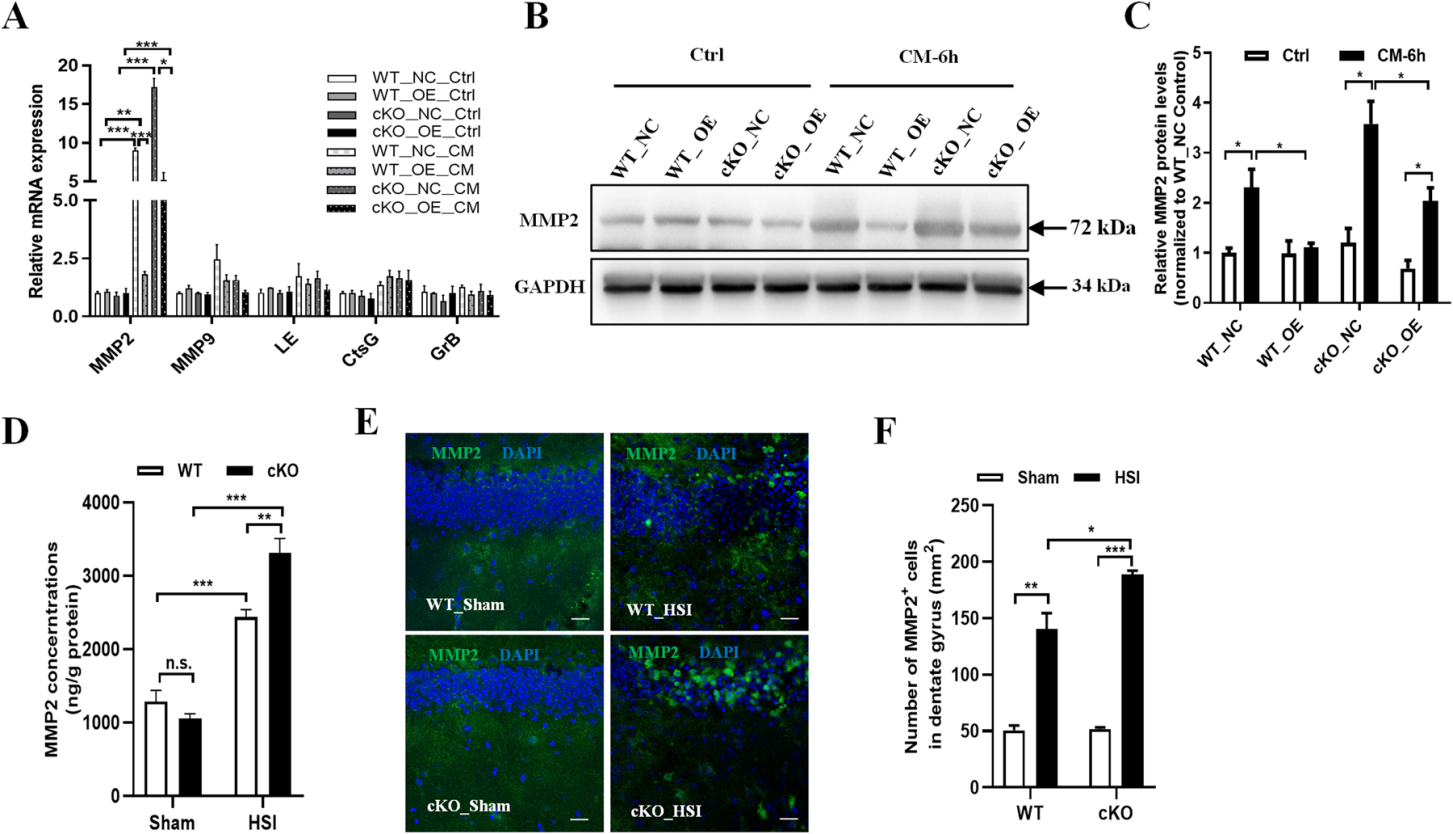
MMP2 is a downstream target of SerpinA3N. **a** qRT-PCR was performed to examine the possible substrates for SerpinA3N in the cultured primary hippocampal neurons that were transduced with lenti-NC or Lenti-*Serpina3n*-OE virus and then treated with CM for 6 h at DIV 7. Only MMP2 mRNA expression levels were negatively regulated by SerpinA3N (*n* = 3). **b**, **c** Western blot assay showed that MMP2 expression was elevated both in SerpinA3N WT and cKO hippocampal neurons following exposure to CM, and MMP2 protein level was much higher in cKO neurons than that of WT neurons in response to CM. GAPDH was served as a loading control (*n* = 3). **d** ELISA assay showed that the content of MMP2 protein in the hippocampus of *Serpina3n* cKO mice was significantly higher than that of WT mice at 4 days after HSI (*n* = 6). **e**, **f** Representative images and quantification of MMP2 (green) immunostaining in the hippocampusof *Serpina3n* WT or cKO mice at 4 days postHSI. Immunostaining assays validated that the MMP2-positive cells were increased in the hippocampus of *Serpina3n* cKO mice than that of WT mice at 4 days after stab injury (*n* = 3). Data are represented as the mean ± SEM; *p < 0.05, **p < 0.01, ***p < 0.001.

SerpinA3N belongs to serpins that functionally binding and inhibiting specific serine protease. A wide range of potential targets for SerpinA3N, including cathepsin G (CtsG), granzyme B (GrB), matrix metalloproteinases (MMPs), and leukocyte elastase (LE), have been characterized^14,15,17,18^. To explore the downstream targets of SerpinA3N in the hippocampus, we examined the expression of all of these substrates for SerpinA3N in cultured *Serpina3n* WT or cKO hippocampal neurons that were transduced with lenti-NC (negative control) or Lenti-*Serpina3n*-OE (overexpression) virus and then treated with CM from LPS-stimulated BV2 cells for 6 h at 7 days in vitro. Under CM treatment, only MMP2 mRNA and protein expression levels were upregulated in cultured neurons. Moreover, both mRNA and protein expression levels of MMP2 were dramatically lower in lenti-*Serpina3n*-OE infected neurons than that of lenti-NC infected neurons (Fig. 4a–c). The same results were also observed in SerpinA3N WT hippocampal neurons that were transduced with Lenti-NC, Lenti-*Serpina3n*-OE, or Lenti-*Serpina3n*-shRNA virus and then treated with CM for 6 h at 7 days in vitro. Overexpression of SerpinA3N did inhibit the expression levels of MMP2, while knockdown of SerpinA3N elevated MMP2 mRNA and protein expression levels following exposure to CM (Supplementary Fig. 2a–c). These findings indicated that MMP2 may be a neural substrate for SerpinA3N.

To further confirm that MMP2 is regulated by SerpinA3N, we performed ELISA assay of MMP2 in the hippocampus of *Serpina3n* WT and cKO mice. We found that the concentration of MMP2 protein in the *Serpina3n* cKO hippocampus was significantly higher than that of WT hippocampus at 4 days after HSI (Fig. 4d). MMP2 immunostaining assay confirmed that the number of MMP2^+^ cells were increased in the *Serpina3n* cKO hippocampus than that of WT hippocampus at 4 days after HSI (Fig. 4e, f). Therefore, our results consistently suggested that MMP2 is a downstream target of SerpinA3N.

### Inhibition of MMP2 reduces neuronal apoptosis and mediates cognitive recovery after hippocampal stab injury

**Fig. 5:**
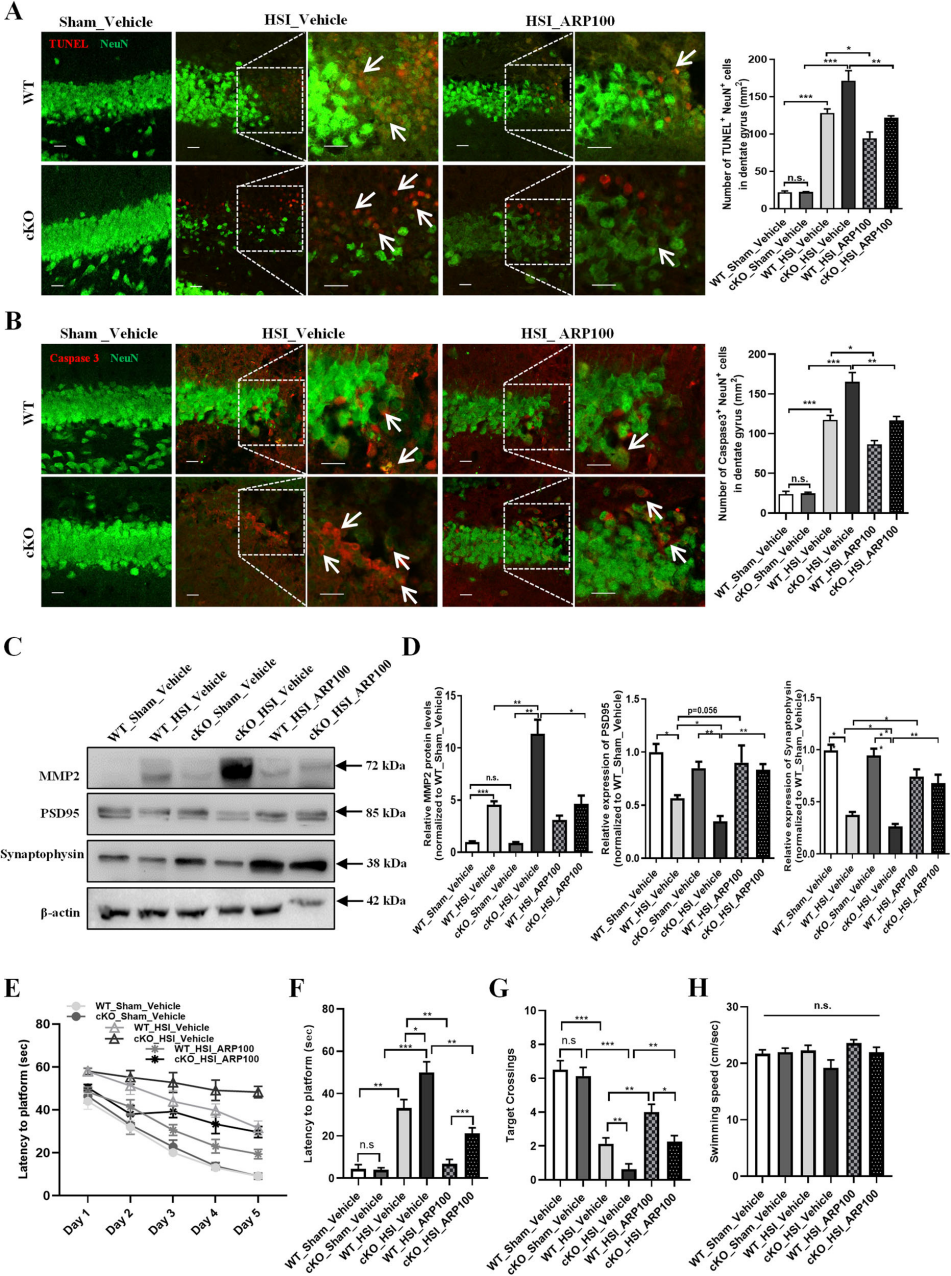
Inhibition of MMP2 reduces neuronal apoptosis and mediates cognitive recovery after HSI. **a** Representative images and quantification of TUNEL (red) immunostaining in the hippocampi of *Serpina3n* WT and cKO mice that received an ARP100 injection daily for four consecutive days. Compared with WT mice, *Serpina3n* cKO mice had a greater degree of neuronal apoptosis in the injured hippocampus. Compared with vehicle control, ARP100 could significantly reduce neuronal apoptosis in both WT and cKO hippocampi after HSI (*n* = 3). **b** Representative images and quantification of caspase 3 (red) immunostaining in the hippocampi of *Serpina3n* WT and cKO mice that received an ARP100 injection daily for four consecutive days. ARP100 did improve neuronal survival in the injured hippocampus (*n* = 3). **c**, **d** Western blot assay showing that PSD95 and Synaptophysin protein levels were significantly lower in the injured *Serpina3n* cKO hippocampus than that of WT hippocampus, ARP100 could enhance the expressions of both PSD95 and synaptophysin in the injured hippocampus (*n* = 4). **e** In the training phase of Morris water maze test, mice from each group showed improved latency to locate the platform, but hippocampal-injured mice exhibited a significant delay to locate the platform compared with sham controls. ARP100 significantly shortened the time to reach the platform (*n* = 8 mice per group). **f** In the probe phase of Morris water maze test, compared with sham groups, both injured *Serpina3n* WT and cKO mice showed a significantly longer latency to locate the platform. ARP100 dramatically reduced the latency to locate the platform for hippocampal-injured mice. **g** In the probe phase of Morris water maze test, ARP100 significantly increased target crossings for injured mice (*n* = 8 mice per group). **h** In the probe phase of Morris water maze test, there was no significant difference in swimming speed between all the groups (*n* = 8 mice per group).

Inhibition of MMP2 has been shown to ameliorate neuronal apoptosis and reduce the complications of brain damage in TBI^19^. Given that MMP2 expression was substantially elevated in injured hippocampus, we thus speculated that an MMP2 inhibitor might be an attractive potential therapeutic for TBI. To test this hypothesis, we applied MMP2 specific inhibitor, ARP100^20,21^, in the neuronal apoptosis analysis and the spatial learning and memory behavioral assays of hippocampal-injured mice. TUNEL and Caspse3 immunostaining assays were firstly performed in the hippocampus of *Serpina3n* WT and cKO mice at 4 days after HSI. We found that treatment with ARP100 significantly reduced neuronal apoptosis in both *Serpina3n* WT and cKO hippocampus after HSI (Fig. 5a, b). Subsequently, we examined the protein levels of PSD95 and synaptophysin in the hippocampus of *Serpina3n* WT and cKO mice after HSI. We found that, to a certain extent, ARP100 could rescue the decreased expressions of both PSD95 and synaptophysin in the injured hippocampus (Fig. 5c, d). These results indicated that targeting MMP2 might restore the impaired cognitive function after HSI.

To further investigate whether inhibition of MMP2 by ARP100 could mediate cognitive recovery after HSI, we conducted the Morris water maze test and the Barnes maze test. In the Morris water maze test, we found that ARP100 could partially rescue the impaired spatial learning and memory after HSI as indicated by significantly reduced latency to locate the platform both in the training phase and in the probe test (Fig. 5e, f) and increased number of target crossings in the probe test (Fig. 5g), but ARP100 did not significantly affect the swimming speed of mice (Fig. 5h). Consistently, in the Barnes maze test, ARP100 significantly decreased the latency to reach the hide box both in the training phase and in the probe test (Supplementary Fig. 3a, b) and increased the times of target crossings in the probe test (Supplementary Fig. 3c), however, no significant difference in total moving distance was observed between all the groups (Supplementary Fig. 3d). Taken together, these data strongly suggested that MMP2 is a key downstream target of SerpinA3N, and MMP2 inhibitor (i.e., ARP100) can serve as a potentially therapeutic agent for the early treatment of neurotrauma.

## Discussion

The observation made in the current study that SerpinA3N protects hippocampal neurons against apoptosis following HSI, does cohere with a previous report of the neuroprotection effect of SerpinA3N in an in vitro cell culture model and an in vivo model of multiple sclerosis (MS), an autoimmune inflammatory and neurodegenerative disease of the central nervous system^13^. In these models of MS, pre-treatment of lymphocytes with SerpinA3N prevents neuronal killing in vitro, while SerpinA3N treatment maintains the integrity of myelin and reduces axonal and neuronal injury in vivo^13^. In this study, we provide the first evidence that SerpinA3N deficiency results in increased neuronal apoptosis and severer cognitive deficits in response to HSI, and inhibition of SerpinA3N prevents cell death of hippocampal neurons after injury.

The role and mechanism of Serpina3n in brain and neurological diseases are still poorly defined. SerpinA3N mRNA is highly expressed in brain, liver, heart, testis, skeletal muscle, lung, thymus, and spleen^16^. It has been demonstrated that SerpinA3N is upregulated in the dorsal root ganglia after nerve injury, and exogenous delivery of SerpinA3N can attenuate neuropathic pain by inhibiting T lymphocytes to release leukocyte elastase^15^. SerpinA3N expression is induced in STAT3-mediated manner in pinealocytes in the rat pineal gland after systemic injection of lipopolysaccharide, however, the function of SerpinA3N under inflammatory conditions is still unknown^22^. In our mouse model of HSI, we found that most SerpinA3N^+^ cells are neurons, and only around 14% SerpinA3N^+^ cells were oligodendrocytes and astrocytes. Moreover, we observed that the knockout of Serpina3n in neurons resulted in increased neuronal apoptosis in response to HSI. We speculate that the main function of SerpinA3N is to protect neurons against apoptosis, it may not directly mediate the inflammatory reaction of astrocytes after hippocampal-injury.

Matrix metalloproteinases (MMPs), a family of zinc-binding proteinase, cleave a wide range of protein substrates in maintaining and remolding the extracellular matrix (ECM)^23–25^, in addition to performing limited cleavage of cytokines, neurotrophins, and cell adhesion molecules^26,27^. Several studies have showed that MMPs, particularly MMP2, is upregulated after stroke, cerebral hypoxia/ischemia, and TBI^28–30^. Activation of MMP2 has been demonstrated as a key mediators of blood-brain barrier (BBB) disruption and up-regulate caspases associated with TBI^31,32^. MMP2 is highly expressed in neurons after TBI, it can cleave the four amino acids at the N-terminus of stromal cell derived factor 1α (SDF-1α) and generate neurotoxic molecular SDF-1, which may induce neuronal death and neurodegeneration^33–36^. Inhibition of MMP2 can prevent the formation of SDF-1 and reduce the apoptosis of hippocampal neurons in vitro^19^. In consistent with this, we found that MMP2 is upregulated in injured hippocampal neurons and the pharmacological MMP2 specific inhibitor ARP100 can dramatically reduce the neuronal apoptosis after TBI. Importantly, we also present here that ARP100 can ameliorate the impaired spatial learning and memory upon loss of *Serpina3n* in mice after HSI. As MMP2 is also highly expressed in astrocytes and participates in the neuroinflammatory cascade that is triggered during stroke^37^ and other neurological diseases^38–40^, we cannot exclude the possibility that ARP100 improves the cognitive performance in hippocampal-injured mice partially by its anti-inflammatory activity. Furthermore, it is well known that the ECM plays a vital role in transducing cellular communication by mediating signaling pathways^41^, whether and how MMP2 inhibition also affect the axon regeneration and synaptic plasticity remains to be investigated.

## Conclusions

The present study strongly suggests that SerpinA3N and MMP2 specific inhibitor may be a new therapeutic agents against neuronal apoptosis and cognitive impairments in neurotrauma. Additional works are needed to confirm the efficiency and safety of targeting the SerpinA3N-MMP2 pathway in treating TBI using animal models considering injury severity, locations, sexes, and ages.

## Materials and methods

### Animals

*Serpina3n*^*flox/flox*^ (JAX stock #027511) and *Emx1-Cre* (JAX stock #005628) mice were obtained from the Jackson Laboratory (Bar Harbor, ME) and crossed with each other to generate the *Serpina3*n^*flox/loxf;Emx1-Cre*^ conditional knockout mouse line (*Serpina3n* cKO). Mice were genotyped by PCR analysis using the genomic DNA isolated from tails. The following primers were used: *Serpina3n*^*f*lox/flox^, forward (5′-TTGACATCCACACTCCCAGA-3′), reverse (5′-CTATCACGGAGGAAGTGCTG-3′), *Emx1-Cre*, transgene forward (5′-AAGGTGTGGTTCCAGAATCG-3′), transgene reverse (5′-CTCTCCACCAGAAGGCTGAG-3′), internal positive control forward (5′-GCGGTCTGGCAGTAAAAACTATC-3′), internal positive control reverse (5′-GTGAAACAGCATTGCTGTCACTT-3′).

### Hippocampal stab injury

To generate an animal model of TBI, two to three-month-old male mice were received a stab wound injury in the hippocampus. Briefly, mice were anesthetized by an intraperitoneal injection of Avertin (200 mg/kg body weight), then positioned in a KOPF stereotaxic apparatus. A cranial window (3 mm in diameter) was opened by a thin blade on the parietal skull, and a hippocampal lesion was made by inserting a bundle of five 26-gauge needles spacing 0.5 mm between each other at the following coordinates determined from the mouse brain in stereotaxic coordinates^42^: 2.5 mm posterior to bregma, 2.3 mm lateral to the sagittal suture, and 2.6 mm below the dura. The sham-operated mice were used as controls and had the same procedures except that no HSI was produced.

### Cell cultures

Primary neuronal culture was conducted as described previously^43^. Briefly, hippocampus was obtained from mice at postnatal day 0 (P0). The hippocampus was minced and digested with 0.25% trypsin (Life) for 10 min at 37 °C, and then terminated with DMEM medium containing 10% FBS. Cell solution was gently triturated and filtered through 70 μm cell strainer. Cells, suspended in neurobasal medium containing B-27 supplemental (Invitrogen, 1:50), Glutamax (Invitrogen, 1:100) and penicillin–streptomycin (Life, 1:100), were seeded into 24-well cell slides coated with PDL. Half medium was changed every three days.

BV2 cells and human embryonic kidney (HEK) 293T cells were cultured in DMEM medium supplemented with 10% FBS and 1% penicillin–streptomycin. BV2 cells were treated with 1 mg/ml LPS (sigma) for 24 h, the cell culture supernatant was then collected as the condition medium (CM) for treating primary cultured hippocampal neurons.

### Western blotting

Primary cultured hippocampal neurons or hippocampal tissues were homogenized in ice-cold RIPA lysis buffer (Beyotime, P0013B) and protease inhibitor PMSF (Beyotime, ST506), and incubated on ice for 30 min, then centrifuged at 13,000 rpm for 15 min at 4 °C. Protein concentrations were determined by the BCA assay kit (PA101-01), and protein samples were separated on 8–12% SDS-PAGE gels and blotted onto polyvinylidene fluoride membrane (Millipore). Membrane was blocked for 60 min in 3% milk, and then incubated overnight at 4 °C with anti-SerpinA3N (1:500 R&D Systems, AF7409), anti-caspase3 (1:500 Abcam, ab13847), anti-PSD95 (1:1000 Abcam, ab18258), anti-synaptophysin (1:1000 Abcam, ab8049), anti-MMP2 (1:1000 Abcam, ab37150), anti-β-actin (1:3000, Sigma, A5441) or anti-GAPDH (1:2000 Beyotime, AF0006) antibody. After washing, membranes were incubated with horseradish peroxidase (HRP)-linked goat anti-mouse, goat anti-rabbit or donkey anti-goat antibody. The immunoreactive bands were detected with the enhanced chemiluminescence reagent (ECL, Pierce) and quantified using Image J Software.

### qRT-PCR

Total RNA was extracted from the cultured neurons or hippocampal tissues with TRIzol (Invitrogen) according to the manufacture’s protocol. RNA quality and concentration were measured by a NanoDrop 2000 (Thermo Fisher). cDNA was reverse-transcribed using the One-Step gDNA Removal and cDNA Synthesis Kit (TransGen Biotech). RT-PCR reactions were performed in triplicate by Hieff^TM^ qPCR SYBR Green Master Mix (YEASEN). *Actb* served as a reference gene. The expression levels of genes were calculated by the ΔΔCT method. All primers used for qRT-PCR are summarized in Supplementary Table 1.

### Enzyme-linked immune sorbent assay (ELISA)

Mouse MMP-2 PicoKine ELISA kit (BOSTER, EK0460) was used for quantitative detection of MMP-2 in cell culture cell culture supernatant or in hippocampal tissues according to the manufacturer’s instructions.

### Immunocytochemistry and immunohistochemistry

Immunocytochemistry and immunohistochemistry were performed according to published protocols^44–46^. For immunostaining cultured neurons, cells were fixed with 4% PFA and blocked in blocking solution (3% BSA containing 0.25% Triton X-100, 0.2% sodium azide). Primary antibody incubation was performed overnight at 4 °C (MAP2 1:1000, Milipore, MAB3418; MMP2; or Caspase3; SerpinA3N). For immunohistochemistry staining, mice were anesthetized with an overdose of Avertin (300 mg/kg body weight) and transcardially perfused with cold PBS (pH 7.4), followed by 4% paraformaldehyde in phosphate buffer (0.1 M, pH 7.4). Brains were harvested and sectioned into 40 μm-thick serial sections. For antibody staining, brain sections were washed in PBS for 10 min upto three times, followed by 1 h RT blocking in blocking solution and overnight incubation with primary antibody (SerinA3N 1:500, R&D Systems; Iba1 1:1000, Wako, 019-19741; NeuN 1:1000, Milipore; GFAP 1:1000, Proteintech, 16825-1; Olig2, Millipore, MAB377; MMP2 1:500, Abcam; Caspase3 1:500, CST). Upon washing, sections were incubated for 2 h at RT with second antibodies conjugated to Alexa Fluor 488 or 568 (Invitrogen). The slides were then observed with a fluorescence microscopy (Zeiss LSM 710 Microscopy).

### TUNEL assay

To detect the neuronal apoptosis, Terminal Deoxynucleotidyl transferase dUTP nick end labeling (TUNEL) staining (In situ cell death detection kit, Beyotime) was performed. Briefly, frozen tissue sections were rinsed with 0.01 M PBS for 5 min and incubated with 2 % BSA containing 0.25% triton X-100 for 30 min at RT. Subsequently, slides were treated with 200 μL TUNEL reaction mixture at 37 °C for 1 h, followed by three washes with 0.01 M PBS. After that, slides were incubated overnight with anti-NeuN (1:1000, milipore, ABN78) antibody at 4 °C. Sections were washed with 0.01 M PBS for 10 min upto three times and then incubated with the secondary antibodies conjugated to Alexa Fluor 488 (1:1000, Life Sciences) at RT.

### Fluoro-Jade C (FJC) staining

FJC staining was performed to stain all degenerating neurons. Brains were sectioned into 20 μm-thick and stained with FJC using a published protocol^47^. In brief, sections were firstly rinsed in a solution containing 1% sodium hydroxide in 80% ethanol for 5 min, followed by 2 min in 70% alcohol and 2 min in distilled water. The sections were then incubated with 0.0001% solution of FJC made in 0.1% acetic acid for 20 min and subsequently rinsed, dried, and mounted in mounting medium.

### Behavioral tests

Mice were kept in groups of 3–5 animals on a 12:12 h light:dark cycle. The Morris water maze test and the Barnes maze test were performed during the light phase as previously described^45,48^. Videos were recorded and analyzed using the behavioral software Smart V3.0.03 (Panlab, Barcelona, Spain). Observers who were in blind, collected primary data.

The Morris water maze is a 120-cm diameter circular tank filled with opaque water using non-toxic white paint. A 13-cm in diameter round platform was hidden 1.5 cm beneath the surface of the water at the center of a given quadrant of the water tank. During the five successive training days, mice were trained with four trails per day. For each trail, the mouse was released from the wall of the water tank and allowed to swim for the acquisition of the platform and to rest on the platform for 20 s within the 60 s trail period. Twenty-four hours after the last acquisition test, the platform was removed and probe trails were conducted. The mice task performance, including swimming speed, tracks, time spent, and entries in each quadrant were recorded.

The Barnes maze is a 122-cm diameter circular platform with 20 evenly spaced holes (2 cm away from the edge, 5 cm in diameter), with only one hole leading to a removable hiding box located directly below the platform. Briefly, for the acquisition of the hiding box, the learning test was conducted for 5 days within 5 min of each trail and times times per day. Immediately after entering the target box, the mouse was allowed to stay there for 30 s. A probe trail was performed for 5 min per animal on the day following the last day of acquisition. Animal tracks and moving speed were recorded.

### Lentiviral constructs

Mouse *Serpina3n* cDNA was amplified from cerebral cortex by PCR and then subcloned into the pCD511B-copGFP (Youbio) lentiviral construct under control of CMV promoter. For knocking down *Serpina3n*, the *Serpina3n* shRNA sequence (5′-GGAGTCAAATTTGTCCCAATG-3′) was inserted into the pCD511B-copGFP (Youbio) plasmid under control of U6 promoter. All vectors were verified by DNA sequencing before use. Lentivirus was produced as described previously^44,49^.

### Statistical analysis

Statistical analysis was performed using SPSS software (SPSS V23; IBM). All data were presented as mean ± SEM, and statistical significance was defined as *p < 0.05, **p < 0.01, and ***p < 0.001 by either unpaired two-tailed students’ *t*-tests or ANOVA with Tukey’s post-hoc tests.

## Supplementary information

Supplementary Figure Legends

Supplementary Figure 1

Supplementary Figure 2

Supplementary Figure 3

Supplementary Table
