# Supplementary material for: SerpinA3N deficiency deteriorates impairments of learning and memory in mice following hippocampal stab injury
Source: Cell Death Discov. 2020 Sep 18;6:88. doi: 10.1038/s41420-020-00325-8 (PMC7501238; doi:10.1038/s41420-020-00325-8)
Supplement: Supplementary file 1 — Supplementary Figure Legends [file 41420_2020_325_MOESM1_ESM.docx]

**Supplementary information**

**Supplementary Figures**

**Supplementary Fig. 1 *Serpina3n* is upregulated in hippocampal neurons following HSI or exposure to CM in vitro.**

**a** Representative images of the co-staining of SerpinA3N, GFAP (a marker of astrocyte), Olig2 (a marker of oligodendrocyte), or Iba1 (a marker of microglia) at 4 days after hippocampal stab injury (n = 8).

**b, c** Verification of increased expression levels of SerpinA3N mRNA (b) and protein (c) in primary hippocampal neurons that were treated with CM for 6hr at DIV 7 (n = 3). GAPDH was served as a loading control in Western blotting.

**d** Representative images and higher fluorescence intensity of SerpinA3N (red) staining in cultured hippocampal neurons that were treated with CM from BV2 cells subjected to LPS exposure (n = 10 neurons per group).

Scale bars, 20 μm. Data are represented as the mean ± SEM; two-tailed *t* test; *p<0.05, **p<0.01, ***p<0.001

**Supplementary Fig. 2 MMP2 is a substrate of SerpinA3N.**

a qRT-PCR was performed to determine the mRNA expressions of MMP2, MMP9, CtsG, LE and GrB in cultured hippocampal neurons that were transduced with lenti-NC, lenti-*Serpina3n*-OE or lenti-*Serpina3n*-shRNA virus and then treated with CM for 6hR at 7 days in vitro. Under CM treatment, MMP2 mRNA expression was down-regulated in lenti-*Serpina3n*-OE infected neurons, but up-regulated in lenti-*Serpina3n*-shRNA transfected neurons (n = 3).

**b** Western blot assay was conducted to validate the result of qRT-PCR analysis. There was a remarkable negative correlation between MMP2 and SerpinA3N (n = 3). GAPDH was used as an internal control, and protein expression was quantified by normalizing to GAPDH.

**c** Representative images and quantification of MMP2 (red) immunostaining in the cultured primary hippocampal neurons that were transduced with lenti-NC, lenti-*Serpina3n*-OE or lenti-*Serpina3n*-shRNA virus and then treated with CM for 6hr at DIV 7. Immunostaining assay validated that the fluorescence intensity of MMP2 was lower in lenti-*Serpina3n*-OE infected neurons but higher in lenti-*Serpina3n*-shRNA transfected neurons (n = 10 neurons per group). Scale bars, 50 μm.

Data are represented as the mean ± SEM; two-tailed *t* test; *p<0.05, **p<0.01, ***p<0.001

**Supplementary Fig. 3 Inhibition of MMP2 enhances cognitive recovery of hippocampal-injured mice.**

**a** In the training phase of Barnes maze test, mice from each group showed improved latency of first entrance into the hiding box. Compared with sham controls, both injured *Serpina3n* WT and cKO mice spent more time to locate the hiding box. ARP100 treatment significantly reduced the latency of first entrance into the hiding box for hippocampal-injured mice (n=8 mice per group).

**b** Compared with vehicle groups, ARP100 treatment decreased the latency of first entrance into the target hole for hippocampal-injured mice (n = 8 mice per group).

**c** Compared with vehicle groups, ARP100 treatment increased the times of target crossings for hippocampal-injured mice (n = 8 mice per group).

**d** In the probe trails, no significant difference in total moving distance was observed between any groups of mice (n = 8 mice per group).

Data are represented as the mean ± SEM; ANOVA with Tukey’s post hoc tests; *p<0.05, **p<0.01, ***p<0.001

**Supplementary Table 1.** Primers used for qRT-PCR.
