# Supplementary figures and images for: SerpinA3N deficiency deteriorates impairments of learning and memory in mice following hippocampal stab injury
Source: Cell Death Discov. 2020 Sep 18;6:88. doi: 10.1038/s41420-020-00325-8 (PMC7501238; doi:10.1038/s41420-020-00325-8)

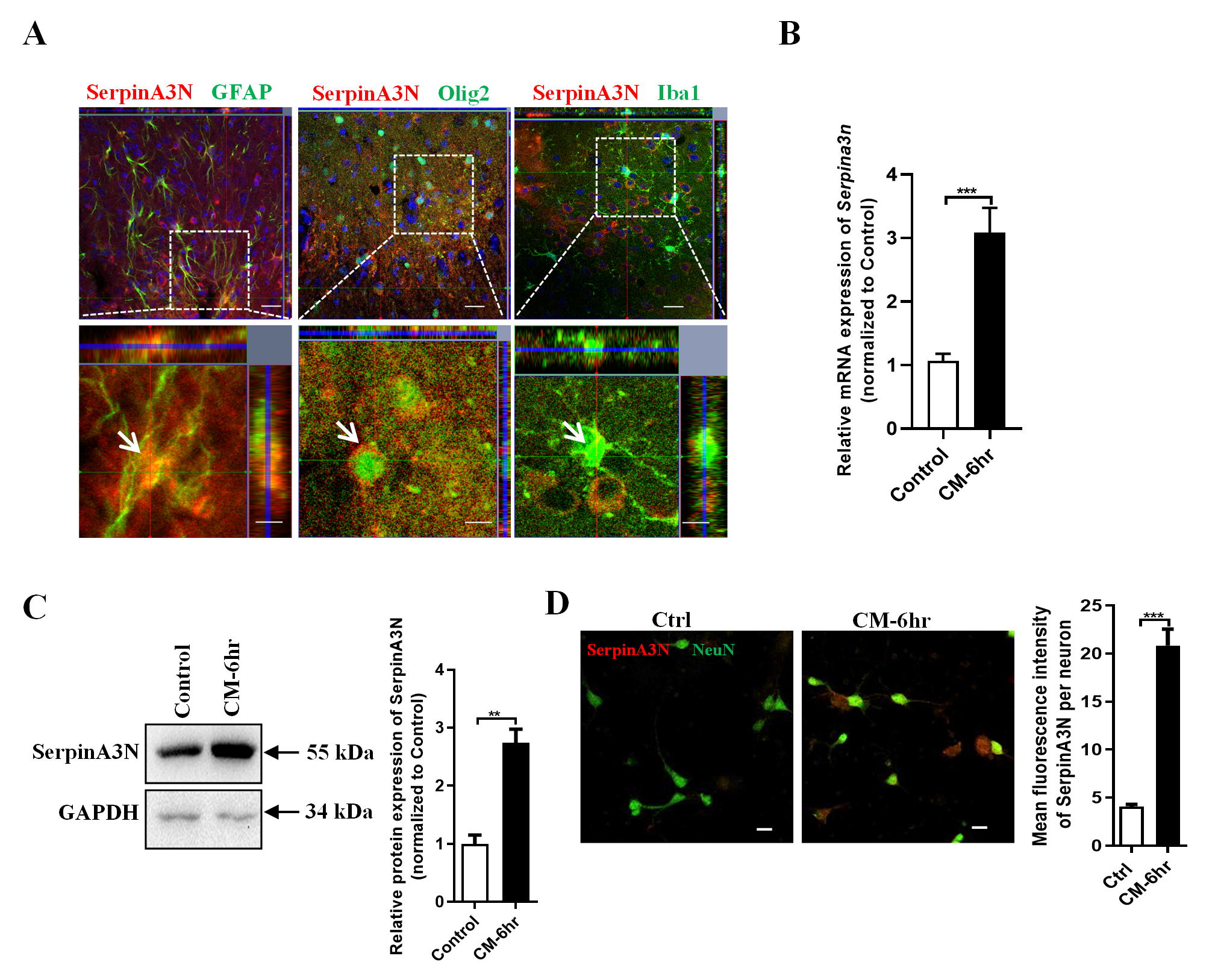

Supplement: Supplementary file 2 — Supplementary Figure 1 [file 41420_2020_325_MOESM2_ESM.tif]

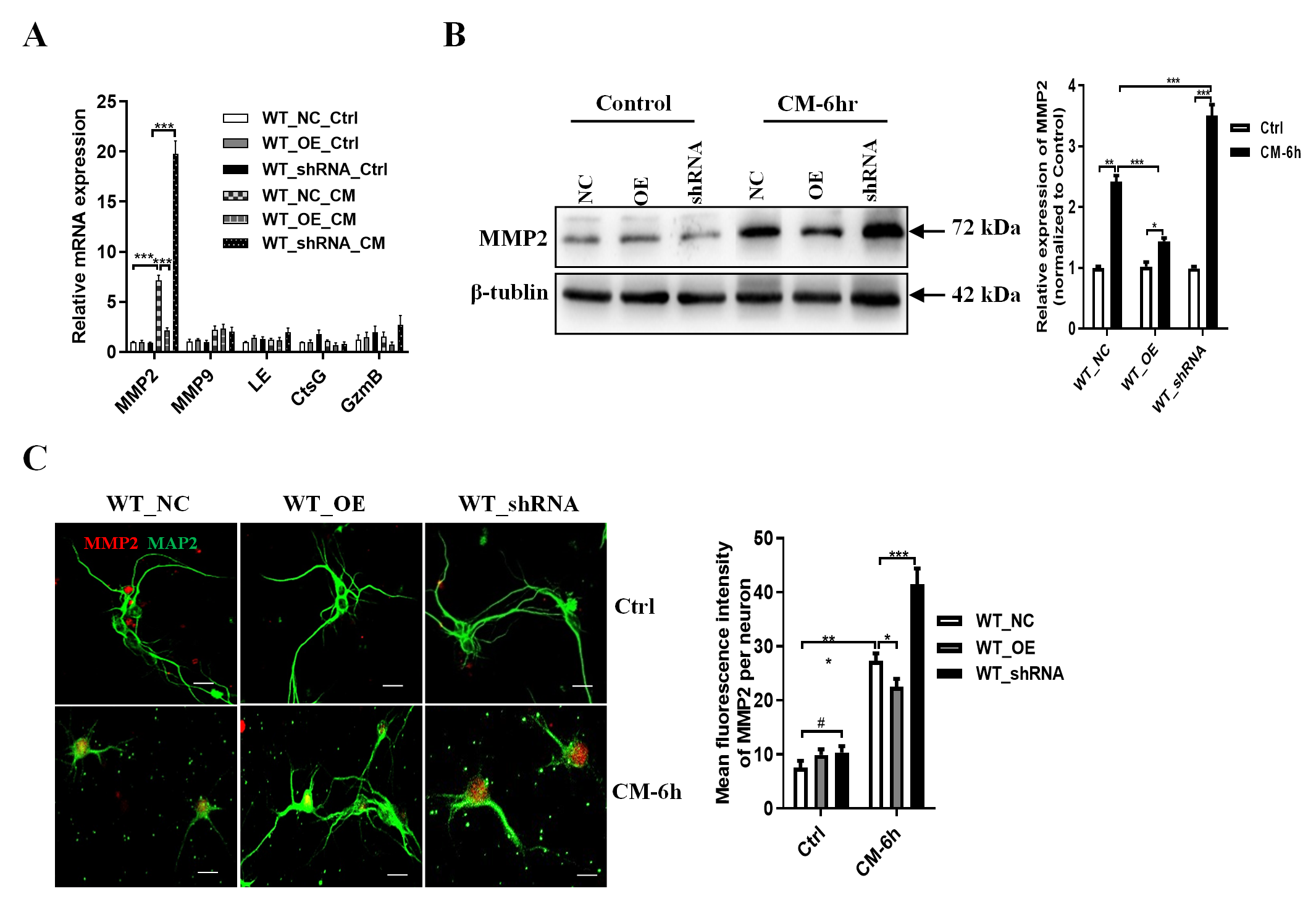

Supplement: Supplementary file 3 — Supplementary Figure 2 [file 41420_2020_325_MOESM3_ESM.tif]

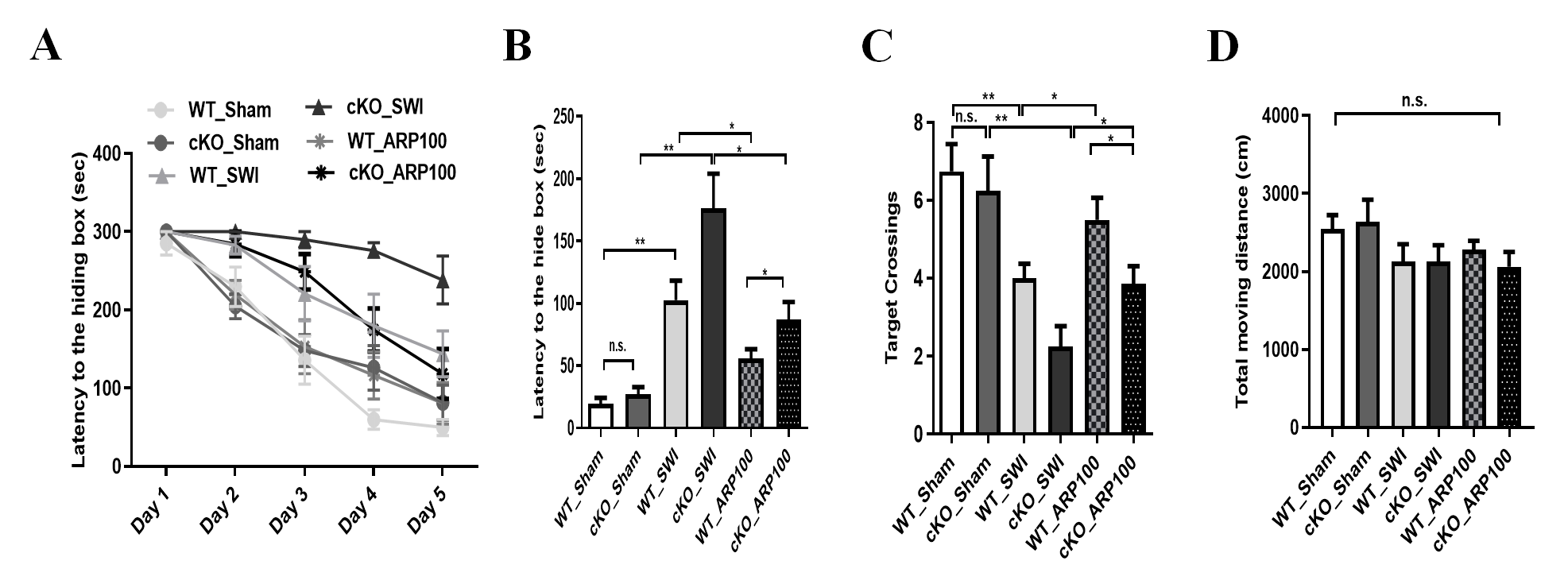

Supplement: Supplementary file 4 — Supplementary Figure 3 [file 41420_2020_325_MOESM4_ESM.tif]
