# Supplementary material for: SerpinA3N deficiency deteriorates impairments of learning and memory in mice following hippocampal stab injury
Source: Cell Death Discov. 2020 Sep 18;6:88. doi: 10.1038/s41420-020-00325-8 (PMC7501238; doi:10.1038/s41420-020-00325-8)
Supplement: Supplementary file 5 — Supplementary Table [file 41420_2020_325_MOESM5_ESM.docx]

**Supplementary Table 1.** Primers used for qRT-PCR.

| **Primer** | **Sequence (5' --> 3')** |
| --- | --- |
| Serpina3n-Forward | CAACCAGAGACCCTGAGGAAGT |
| Serpina3n -Reverse | AGGACATCCTCCAGGCTGTAGT |
| MMP2-Forward | CAAGGATGGACTCCTGGCACAT |
| MMP2-Reverse | TACTCGCCATCAGCGTTCCCAT |
| MMP9-Forward | GCTGACTACGATAAGGACGGCA |
| MMP9-Reverse | TAGTGGTGCAGGCAGAGTAGGA |
| LE-Forward | AGTGTGAACGGACTGGTGCTGT |
| LE-Reverse | GTCTCCTTCAGGTGGACTTCCA |
| CtsG-Forward | AGTCCAGAAGGGCTGAGTGCTT |
| CtsG-Reverse | GCACTGTGATGAGTTGCTGGGT |
| GrB-Forward | CAGGAGAAGACCCAGCAAGTCA |
| GrB-Reverse | CTCACAGCTCTAGTCCTCTTGG |
| Actin-Forward | GCCACCAGTTCGCCATGGAT |
| Actin-Reverse | CATCACACCCTGGTGCCTAG |
